# Supplementary material for: Direct functionalization of methane into ethanol over copper modified polymeric carbon nitride via photocatalysis
Source: Nat Commun. 2019 Jan 31;10:506. doi: 10.1038/s41467-019-08454-0 (PMC6355835; doi:10.1038/s41467-019-08454-0)
Supplement: Supplementary file 1 — Supplementary Information [file 41467_2019_8454_MOESM1_ESM.pdf]

Supplementary Information

Direct functionalization of methane into ethanol over copper modified polymeric carbon nitride via photocatalysis

Zhou *et al.*

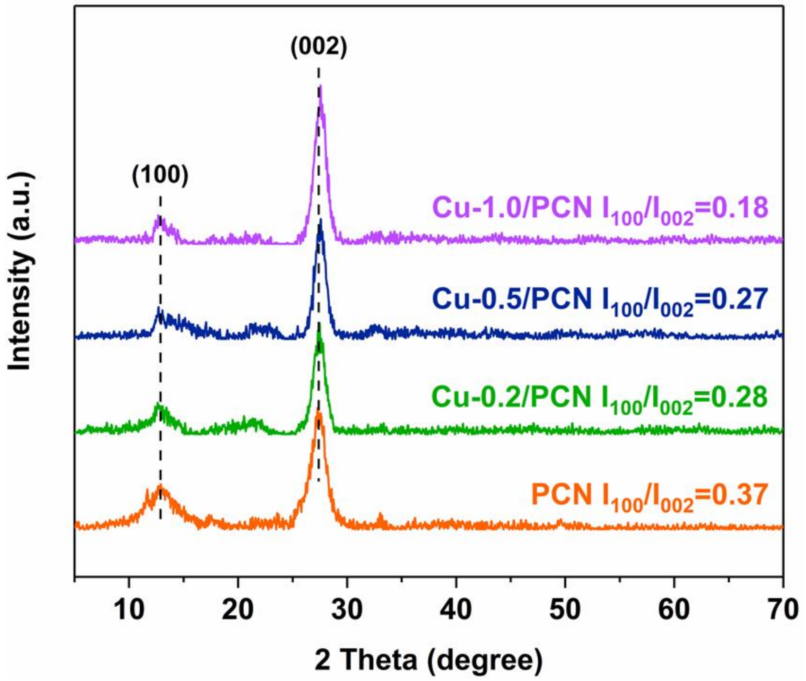

Supplementary Figure 1. XRD patterns of PCN and Cu-X/PCN. The interfacial stacking periodicity of PCN had been destructed with Cu introduction.

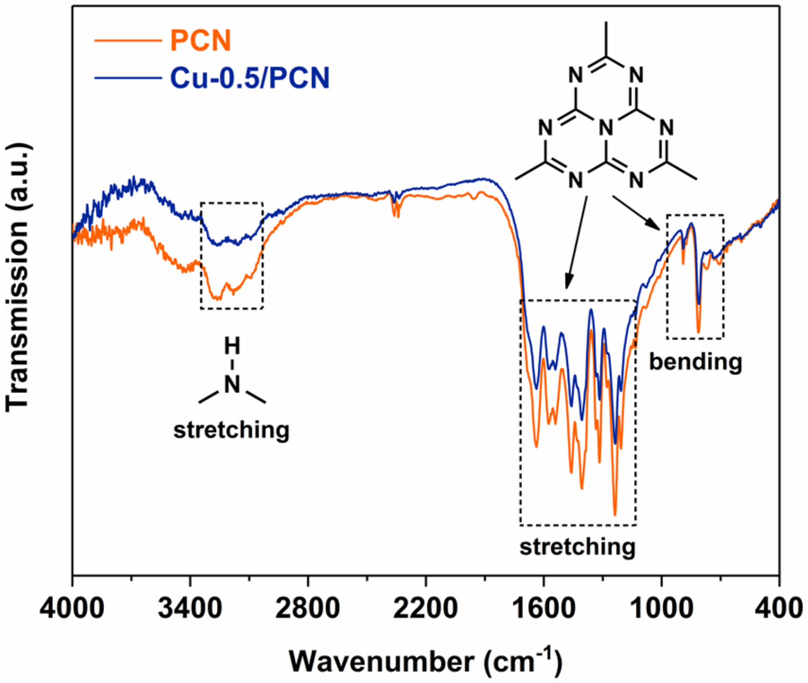

Supplementary Figure 2. FTIR spectra of PCN and Cu-0.5/PCN. No distinct changes of the band positions occurred after Cu introduction, confirming that modification of PCN exerted a negligible influence on its basic structure.

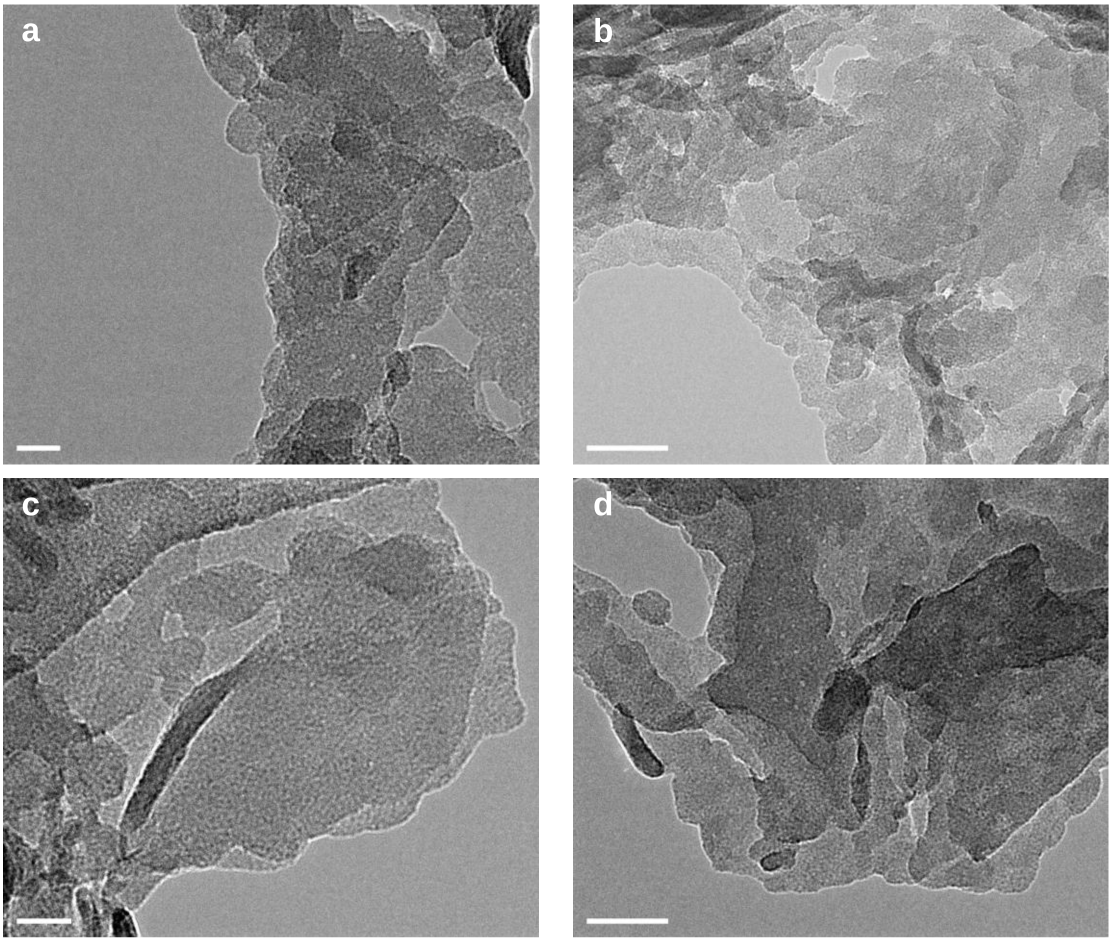

Supplementary Figure 3. Morphology of the materials. TEM images of (a, b) PCN and (c, d) Cu-0.5/PCN. Scale bar: (a, c) 20 nm and (b, d) 50 nm. No more than the layered structure of PCN was observed with Cu incorporation.

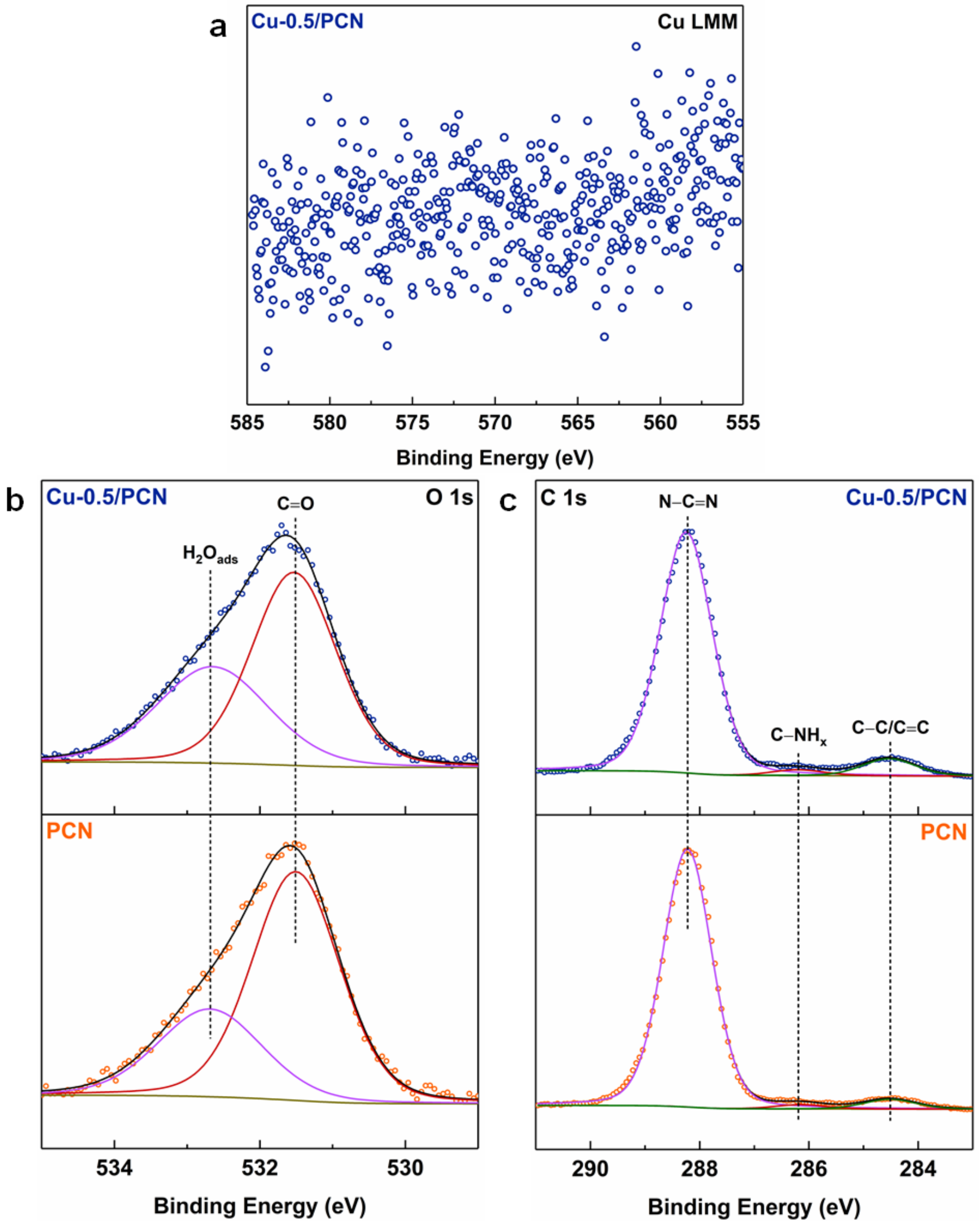

Supplementary Figure 4. Valence state and bonding situation analysis. (a) Cu LMM XPS spectrum of Cu-0.5/PCN; (b) O 1s XPS spectra of PCN and Cu-0.5/PCN; (c) C 1s XPS spectra of PCN and Cu-0.5/PCN. No peak of Cu LMM was detected but noises, meaning that Cu^0 could not be told from Cu^I. Neither O 1s nor C 1s XPS spectra exhibited any shift, excluding the situation of Cu oxides or Cu coordination with C atoms.

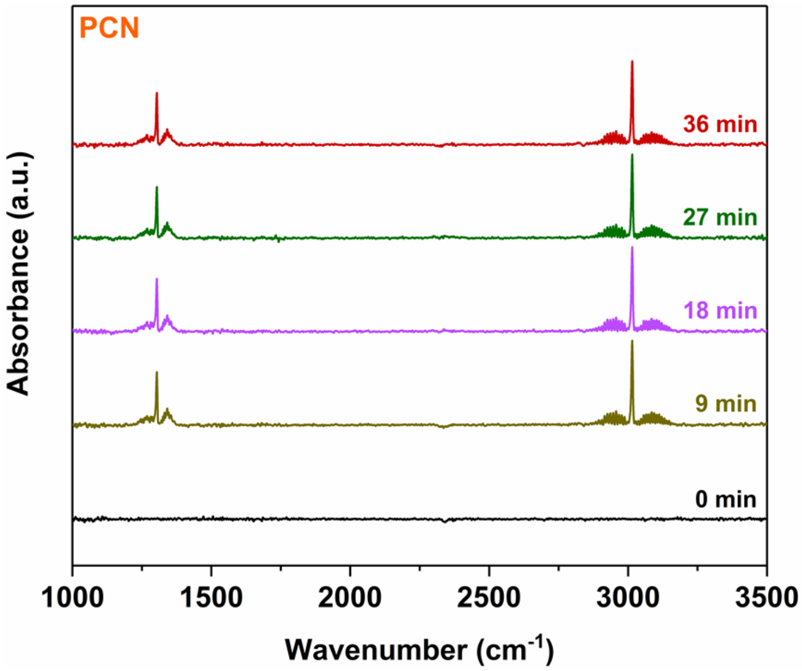

Supplementary Figure 5. *In situ* IR spectra of methane adsorption on PCN. Intensities of the bands originated from methane adsorption kept unchanged, indicating weaker interaction with respect to Cu-0.5/PCN.

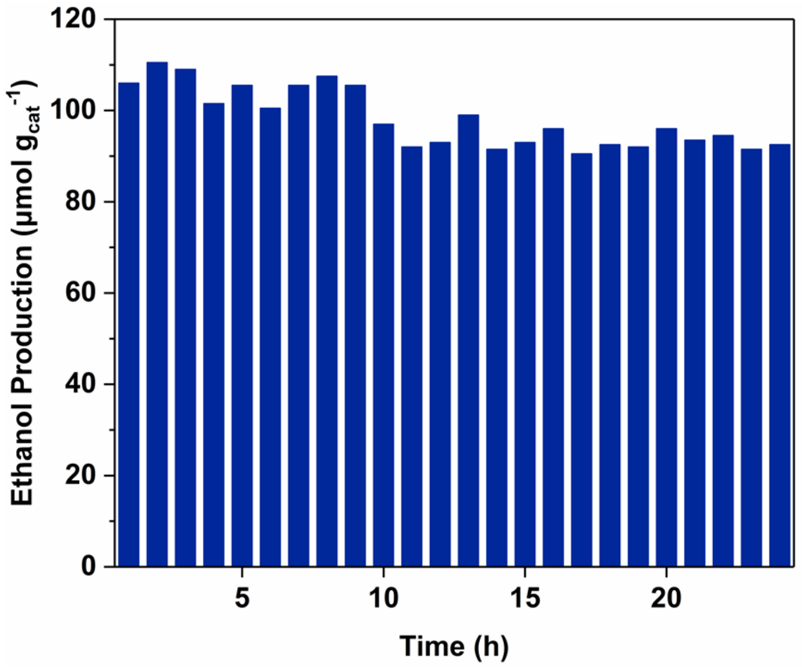

Supplementary Figure 6. Time course of photocatalytic methane-to-ethanol conversion over Cu-0.5/PCN for 24 h. Ethanol production decayed slightly over 24 h of testing.

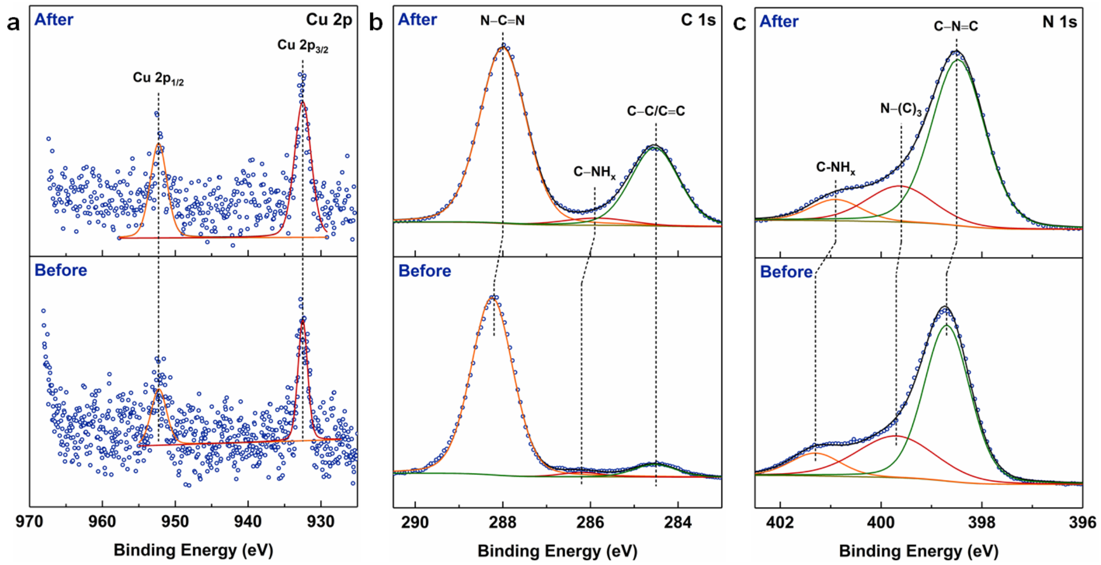

Supplementary Figure 7. Valence state and bonding situation analysis before and after cycling tests. Cu 2p (a), C 1s (b), and N 1s (c) XPS spectra of Cu-0.5/PCN. From the Cu 2p XPS spectra, the mixed-valence state remained unchanged after photocatalytic tests, indicating that the oxidized Cu species from H_2O_2 decomposition were reduced by the photo-induced electrons. In the C 1s XPS spectra, the peak at 284.5 eV became obvious, corresponding to coke deposition and agreeing with the ethanol production decay on Cu-0.5/PCN. The binding energy of all the peaks in the N 1s spectra was smaller in comparison to the fresh photocatalyst, which implied the increasing of the electron density of all kinds of N atoms.

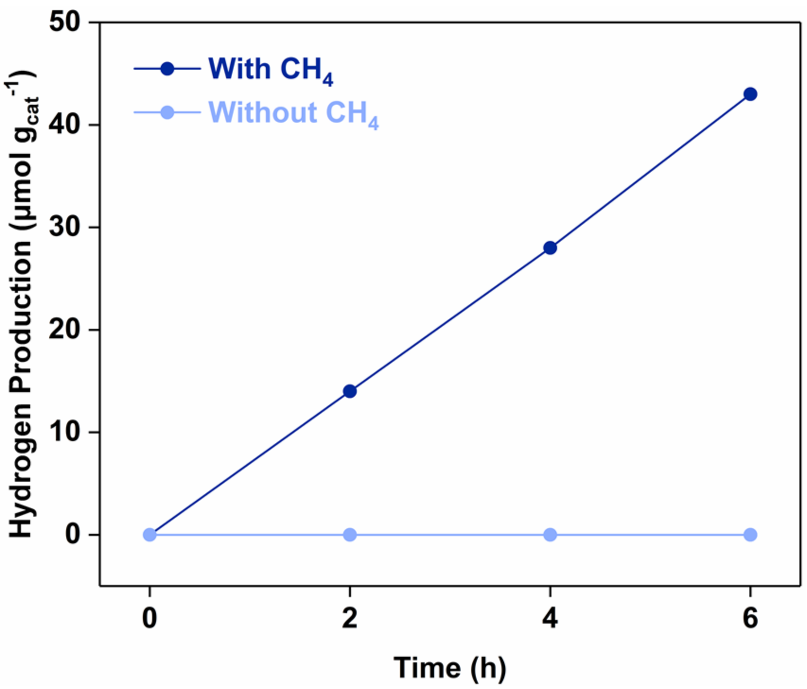

Supplementary Figure 8. Photocatalytic hydrogen evolution over Cu-0.5/PCN with and without methane. No H_2 was detected in the methane-free experiment, excluding H_2 evolution from photocatalytic H_2O splitting.

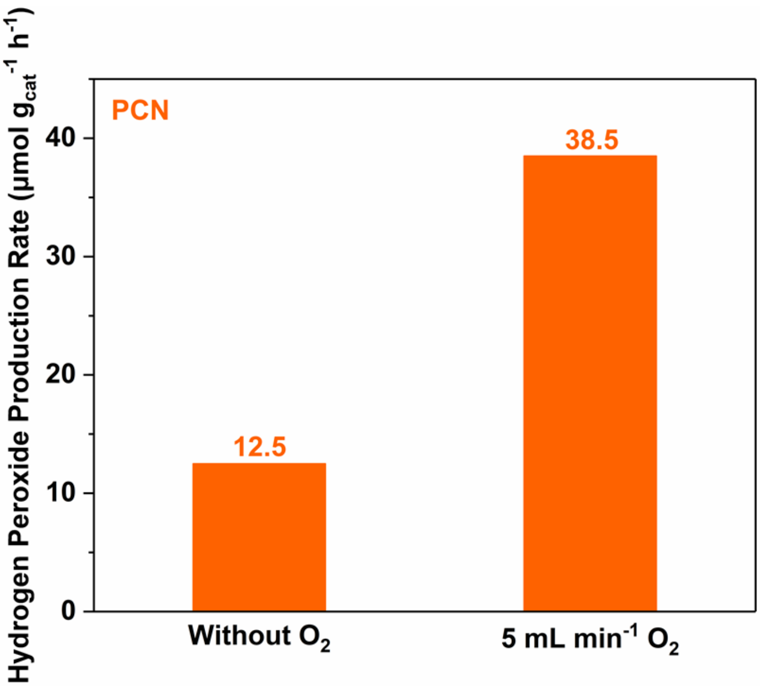

Supplementary Figure 9. Photocatalytic hydrogen peroxide production over PCN with and without oxygen. Introducing O_2 into the system facilitated H_2O_2 production.

Supplementary Table 1. Element content of PCN and Cu-0.5/PCN from XPS analyses.

Atomic %	C	N	O	Cu
**PCN**	39.55	54.63	5.82	0
**Cu-0.5/PCN**	39.27	52.35	8.07	0.31
